# Prognosis and influencing factors of ER-positive, HER2-low breast cancer patients with residual disease after neoadjuvant chemotherapy: a retrospective study

**DOI:** 10.1038/s41598-024-62592-0

**Published:** 2024-05-23

**Authors:** Lingfeng Tang, Linshan Jiang, Xiujie Shu, Yudi Jin, Haochen Yu, Shengchun Liu

**Affiliations:** 1 Department of Breast and Thyroid Surgery, The First Affiliated Hospital of Chongqing Medical University, 1 Youyi Rd, Yuanjiagang, Yuzhong district, Chongqing, 400016 China; 2 Department of Pathology, Chongqing University Cancer Hospital, Chongqing, China

**Keywords:** Neoadjuvant chemotherapy, Breast cancer, Estrogen receptor, HER2-low, Prognosis, Breast cancer, Oncology, Risk factors

## Abstract

Previously, we found that patients with estrogen receptor (ER)-positive, HER2-low breast cancer are resistant to neoadjuvant chemotherapy (NACT) and have worse outcomes than those who achieve pathological complete response (pCR) after NACT. This study aimed to investigate the prognosis and influencing factors in these patients. A total of 618 patients with ER-positive breast cancer who received standard thrice-weekly NACT were enrolled, including 411 patients with ER-positive, HER2-low breast cancer. Data on the clinicopathological features of these patients before and after NACT were collected. Univariate and multivariate Cox regression analyses were used to identify the independent factors affecting 5-year disease-free survival (DFS). Among the ER-positive, HER2-low patients, 49 (11.9%) achieved a pCR after NACT. A significant difference in survival was observed between patients with and without residual disease after NACT. Additionally, changes in immunohistochemical markers and tumor stages before and after NACT were found to be significant. According to univariate and multivariate analyses, cN_stage (*P* = 0.002), ER (*P* = 0.002) and Ki67 (*P* = 0.023) expression before NACT were significantly associated with 5-year DFS, while pT_stage (*P* = 0.015), pN_stage (*P* = 0.029), ER (*P* = 0.020) and Ki67 (*P* < 0.001) levels after NACT were related to 5-year DFS in ER-positive, HER2-low patients with residual disease. Our study suggested that high proliferation, low ER expression and advanced stage before and after NACT are associated with a poor prognosis, providing useful information for developing long-term treatment strategies for ER-positive, HER2-low breast cancer in patients with residual disease in the future.

## Introduction

Breast cancer, the most frequently diagnosed cancer in women, is characterized by distinct pathological subtypes based on the expression of hormone receptors (HRs) and human epidermal growth factor receptor 2 (HER2)^1^. ERBB2 can activate multiple tumor-related signaling pathways, leading to rapid disease progression and a poor prognosis^2–4^. In recent years, anti-HER2 agents have significantly improved outcomes for patients with HER2-enriched breast cancer^5,6^. Furthermore, novel antibody‒drug conjugates (ADCs) have been developed to treat HER2-low (immunohistochemistry (IHC) 1 + or IHC 2 +, fluorescence in situ hybridization (FISH) nonamplified) breast cancer^7,8^. Based on the analysis of clinical characteristics and chemosensitivity, it has been suggested that HER2-low breast cancer, accounting for 70% of all cases, may represent a subtype distinct from HER2-negative (IHC 0) breast cancer^9–12^.

Neoadjuvant chemotherapy (NACT), utilized before surgery, is primarily used for the management of locally advanced breast cancer^13^. Generally, among various breast cancer subtypes, estrogen receptor (ER)-positive breast cancer exhibits poor responsiveness to chemotherapy^14^. Additionally, a comprehensive analysis of several large clinical studies demonstrated that HER2-low status is associated with increased resistance to NACT in HR-positive breast cancer patients (13.7% of HER2-low patients vs. 19.8% of HER2-negative patients, *P* = 0.014)^11^. Consistent with these findings, our previous study revealed that compared with ER-positive, HER2-negative breast cancer, ER-positive, HER2-low breast cancer displays greater resistance to chemotherapy. The pathological complete response (pCR) rate among ER-positive, HER2-low breast cancer patients was nearly half that of ER-positive, HER2-negative breast cancer patients (*P* = 0.014)^12^. Moreover, de Nonneville et al. reported similar associations between ER-positive, HER2-low status and achieving a pCR after NACT (10% of HER2-low patients vs. 16% of HER2-negative patients, *p* = 0.046)^10^.

Compared with HER2-negative status, HER2-low status was not found to correlate with a worse long-term prognosis. However, patients with residual disease exhibited a worse prognosis than those who achieved pCR following NACT. The aim of this study was to identify the factors influencing recurrence and metastasis among patients with ER-positive, HER2-low breast cancer after completing standard treatment.

## Materials and methods

### Patients

**Figure 1:**
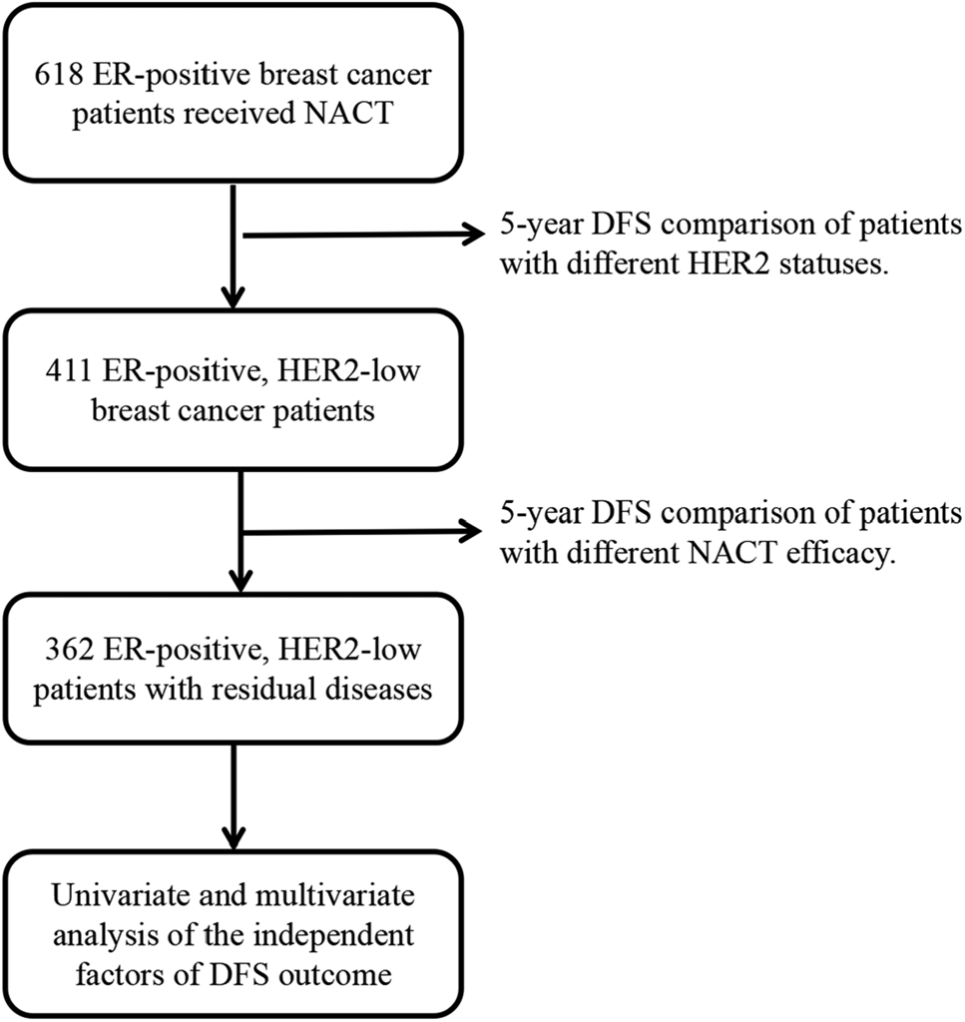
Flow-chart shows the process of including patients in the study. ER, estrogen receptor; NACT neoadjuvant chemotherapy; DFS, disease-free survival.

Medical records of 618 ER-positive breast cancer patients treated from 1 January 2012 to 31 December 2018 were retrieved from clinical databases of the First Affiliated Hospital of Chongqing Medical University. This retrospective study was approved by the Ethics Committee of the First Affiliated Hospital of Chongqing Medical University (No. 2020-202). All procedures were performed in accordance with relevant guidelines. Inclusion criteria: (I) female; (II) performed neoadjuvant chemotherapy; (III) invasive breast cancer; (IV) ER-positive status; and (V) no anti-tumor treatment before NACT. Exclusion criteria: (I) metastasis at diagnosis; (II) other primary tumors; (III) bilateral breast cancer; and (IV) incomplete data. Finally, a total of 618 eligible patients were included in this study (Fig. 1).

All histological specimens were paraffin-embedded and evaluated by two skilled pathologists. This article does not refer to the privacy of patients, so informed consent was exempted. All data were fully anonymized before we accessed them. The authors were not provided with information that could identify individual participants during or after data collection.

### Clinicopathologic analysis

**Figure 2:**
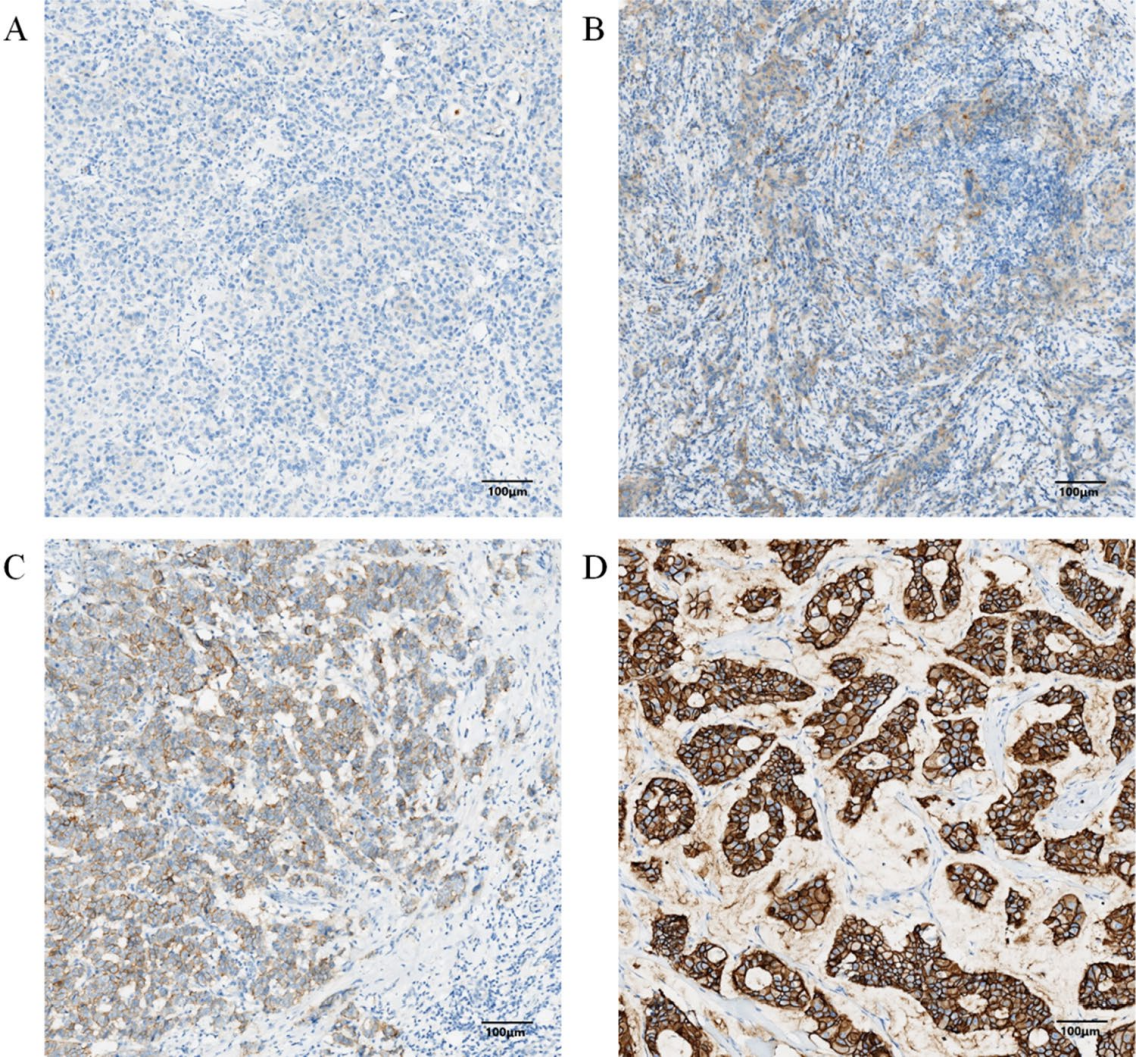
Representative photomicrographs of HER2 in immunohistochemical sections in breast cancer. (**A**) HER2-negative (× 100 magnification). (**B**) HER2-low expression (IHC scores of 1 +) (× 100 magnification). (**C**) HER2-low expression (IHC scores of 2 + with FISH non-amplified) (× 100 magnification). (**D**) HER2-enriched (× 100 magnification).

In order to comprehensively analyze the factors influencing the outcomes of patients, we collected the general information (medical history, age, menopausal status, NACT regimens), pre-NACT clinicopathologic information (stage_cT, stage_cN, pre-ER status, pre-progesterone receptor (pre-PgR) status, pre-Ki67 index) and post-NACT clinicopathologic information (stage_pT, stage_pN, post-ER status, post-PR status, post-Ki67 index). Clinical assessments of the breast, including pre-NACT lymph node status (stage_cN), tumor size (stage_cT) depended on MRI or breast ultrasonography. The pathological diagnosis after the operation would provide us with the data of stage_pN and stage_pT. The pre-ER, pre-PgR, HER2 and pre-Ki67 index were evaluated by IHC on the samples obtained by core needle biopsy. While the post-ER, post-PgR and post-Ki67 index were evaluated by IHC on the samples obtained by operation. The HER2-negative group consisted of the breast cancer patients with a completely negative HER2 staining (IHC score of 0), the HER2-low group consisted of the breast cancer patients with low level of HER2 expression (IHC scores of 1 + and 2 + with FISH nonamplified) and the HER2-enriched group consisted of the breast cancer patients with high level of HER2 expression (IHC scores of 3 + and 2 + with FISH amplified) (Fig. 2). Samples with more than 1% of cells positive for ER/PgR expression were identified ER-positive/PgR-positive. The Ki67 index was defined as the percentage of the total number of tumor cells (at least 1000) with nuclear staining over 10 high powered fields (× 40). RECIST criteria is the standard for the clinical response evaluation^15^.

### Treatment

The NACT criteria for breast cancer patients were as follows: patients with the advanced stage disease, such as patients with axillary lymph node metastasis or large mass or invasion of skin and chest wall. The patients who had a strong desire to conserve breast after operation but did not meet the indication of breast conserving surgery when diagnosed also met the criteria. NACT was performed in accordance with the local protocol and international guidelines of the current year. The treatments were predominantly anthracycline and taxane. The TEC (docetaxel 75 mg/m^2^, epirubicin 75 mg/m^2^, and cyclophosphamide 500 mg/m^2^) or EC (epirubicin 75 mg/m^2^, and cyclophosphamide 500 mg/m^2^) NACT regimens were administered every 3 weeks. After diagnosis, all patients started the first cycle of NACT in a week and received full cycle of NACT regimens (TEC 6 cycles; EC-T 8 cycles) we evaluated the clinical response.

After a series of standard treatments, such as chemotherapy, radiotherapy and surgery, all patients would take up the standard 5-year endocrine therapy (patients who conform the menopause criteria treated with selective non-steroidal aromatase inhibitors, and others treated with estrogen receptor antagonist).

### Objective

For all patients enrolled, mastectomy or breast conserving surgery plus axillary lymphadenectomy was the basic surgical treatment after full cycle NACT. Two skilled pathologists blindly and independently diagnosed all resected breast and lymph node specimens. Then, pCR was defined as no residual invasive cancer in the breast or evidence of disease in the axillary lymph nodes (ypT0ypN0) after NACT. In our study, the patients without achieving pCR after NACT were screened for follow-up analysis. Next, disease-free survival (DFS, defined as the time from surgery to any relapse, secondary malignancy, or death from any cause) was the endpoint in this trial.

### Statistical methods

Statistical analysis was performed by SPSS (Version 25.0). Categorical variables were compared using the chi-squared test or Fisher’s exact test. Then, follow-up was estimated by using the Kaplan–Meier method and the COX regression analysis was utilized to find the influencing factors of DFS. The two-sided log-rank test was used to compare results between groups. The intolerant abilities of the regression analysis results were assessed by calculating the area under the receiver operating characteristic (ROC) curve. *P* < 0.05 was defined as statistically significance.

### Ethical approval

All procedures were performed in accordance with relevant guidelines. Ethical approvals for the study were obtained from the Ethics Committee of the First Affiliated Hospital of Chongqing Medical University (No. 2020-202).

### Informed consent

Informed consents were waived due to the retrospective nature of this study. We declared that patient data was maintained with confidentiality and all the process complies with the requirements of the Ethics Committee of the First Affiliated Hospital of Chongqing Medical University.

## Results

### Survival comparison of ER-positive breast cancer patients

**Figure 3:**
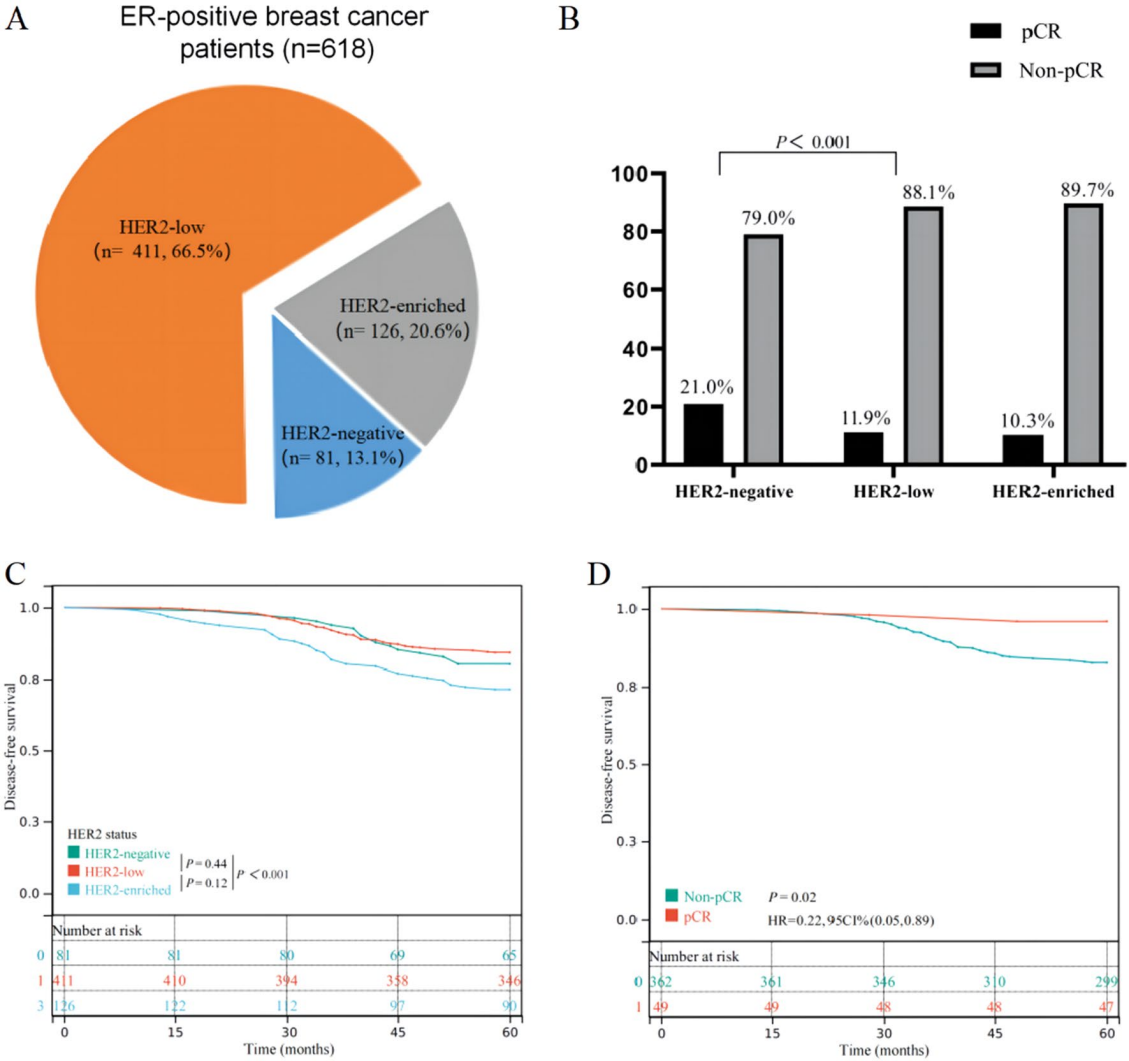
(**A**) Modified classification of breast cancer based on HER2 status. (**B**) Comparison of pathological complete response rates for different HER2 status tumors. Kaplan–Meier survival analyses for 5-year disease-free survival based on (**C**) HER2 status and (**D**) NACT efficacy.

Our study included 618 patients with ER-positive breast cancer who received NACT. Among these patients, 81 were HER2 negative, 411 were HER2-low, and 126 were HER2-enriched (Fig. 3A). The pCR rate of HER2-enriched patients who did not receive targeted therapy was the lowest (10.3%), followed by HER2-low patients (11.9%) (Fig. 3B). The median follow-up periods for HER2-negative, HER2-low, and HER2-enriched patients were 81, 85, and 77 months, respectively. Next, we compared patients’ outcomes based on HER2 status (HER2-negative group vs. HER2-low group vs. HER2-enriched group). As shown in Fig. 3C, HER2-enriched patients had the worst 5-year disease free survival (DFS), while patients with HER2-negative and HER2-low statuses showed no significant difference in outcome (*P* = 0.440). Among the 411 HER2-low patients, 49 (11.9%) achieved pCR after NACT, whereas the remaining 382 (88.1%) had residual diseases. The outcome comparison between the two groups (pCR group vs. non-pCR group) also showed that patients who achieved pCR had a significant better 5-year DFS (*P* = 0.020) (Fig. 3D). Consequently, we enrolled the non-pCR patients with poor outcomes in the followed study (n = 362).

### Clinicopathological characteristics of patients

We collected pre- and post-NACT comprehensive clinicopathological data of ER-positive, HER2-low patients with residual diseases. As shown in Table 1, a total of 362 patients were identified (mean age 49.0 years (range 20–73 years)) and nearly half of the patients (43.1%) met the menopausal standard. In addition, the comparison of pre- and post-NACT data illustrated a significant reduction in disease stages, with a noticeable decrease in the number of patients with large tumors (25.7–6.4%) and half of the cases having local tumors with a diameter of less than 2 cm after NACT (49.1%). Furthermore, the rate of lymph node metastasis decreased after NACT (75.1–67.4%). In terms of routine pathological indicators of breast cancer, such as ER, PR, and Ki67 status, NACT was found to significantly reduce the proliferation index and 27 patients (7.5%) became ER-negative after chemotherapy.

**Table 1:** Baseline clinicopathological characteristics of ER-positive, HER2-low patients with residual diseases.

Characteristic	Pre-chemotherapy	Post-chemotherapy
Age (years)		
< 45	120 (33.1)	
≥ 45	242 (66.9)	
Menopausal status		
Premenopausal	206 (56.9)	
Postmenopausal	156 (43.1)	
T stage		
T1	19 (5.2)	178 (49.1)
T2	250 (69.1)	161 (44.5)
T3/T4	93 (25.7)	23 (6.4)
N stage		
N0	93 (24.7)	118 (32.6)
N1	204 (56.4)	160 (44.2)
N2/N3	65 (17.9)	84 (23.2)
Histological grade		
I/II	248 (68.5)	274 (68.5)
III	114 (31.5)	88 (31.5)
ER status		
Negative		27 (7.5)
(1, 10]	33 (9.1)	20 (5.5)
(10, 40]	74 (20.4)	41 (11.3)
(40, 70]	136 (37.6)	86 (23.8)
> 70	119 (32.9)	188 (51.9)
PgR status		
Negative	83 (22.9)	112 (30.9)
(0, 50]	131 (36.2)	138 (38.2)
> 50	148 (40.9)	112 (30.9)
Ki-67(%)		
≤ 20	133 (36.7)	217 (59.9)
(20, 50]	158 (43.7)	106 (29.3)
> 50	71 (19.6)	39 (10.8)
NACT regimens		
TEC	321 (88.7)	
EC-T	41 (11.3)	

*ER* estrogen receptor, *PgR* progesterone receptor, *NACT* neoadjuvant chemotherapy.

### The pre-NACT factors affecting the outcomes

To begin with, we conducted an analysis of the data collected prior to the NACT and its impact on patient outcomes. Through univariate analysis, the influence of stage_cN (*P* = 0.002), pre-ER status (*P* < 0.001), pre-Ki67 index (*P* = 0.011) on prognosis were studied in the multivariate analysis. We found that stage_cN (*P* = 0.002), pre-ER status (*P* = 0.002), pre-Ki67 index (*P* = 0.023) were independent factors affecting DFS in ER-positive, HER2-low patients with residual diseases (Table 2).

**Table 2:** Univariate and multivariate analyses of 5-year recurrence and patients’ pre-NACT characteristics.

Characteristics	Univariate analysis HR (95% CI)	*P* value	Multivariate analysis HR (95% CI)	*P* value
Age, years (≥ 45 vs. < 45)	0.776 (0.467–1.291)	0.489		
Menopausal status (Postmenopausal vs. Premenopausal)	0.881 (0.683–1.136)	0.329		
cT stage		0.837		
cT1	1 (reference)			
cT2	0.816 (0.293–2.272)			
cT3/cT4	0.726 (0.241–2.187)			
cN stage		**0.002**		**0.002**
cN0	1 (reference)		1 (reference)	
cN1	1.156 (0.592–2.258)		1.245 (0.631–2.455)	
cN2/cN3	2.847 (1.400–5.787)		2.999 (1.456–6.176)	
Histological grade (III vs. I/II)	1.440 (0.869–2.385)	0.157		
pre-ER status		** < 0.001**		**0.002**
(1, 10]	1 (reference)		1 (reference)	
(10, 40]	0.234 (0.105–0.522)		0.293 (0.131–0.657)	
(40, 70]	0.293 (0.153–0.562)		0.352 (0.182–0.681)	
> 70	0.215 (0.105–0.440)		0.297 (0.142–0.621)	
pre-PgR status		0.467		
Negative	1 (reference)			
(0, 50]	0.803 (0.433–1.487)			
> 50	0.675 (0.362–1.259)			
pre-Ki67(%)		**0.011**		**0.023**
≤ 20	1 (reference)		1 (reference)	
(20, 50]	2.529 (1.310–4.885)		2.332 (1.191–4.566)	
> 50	2.774 (1.324–5.808)		2.680 (1.258–5.710)	
NACT regimens (EC-T vs. TEC)	0.758 (0.374–1.535)	0.441		

*DFS*, disease-free survival, *HR* hazard ratio, *CI* confidence interval, *NACT* neoadjuvant chemotherapy, *pre-ER* estrogen receptor before NACT, *pre-PgR* progesterone receptor before NACT, *pre-Ki67* Ki67 index before NACT.

Bold values indicates statistical significance (*P* < 0.05).

### The post-NACT factors affecting the outcomes

Similarly, the analysis of the pathological data from surgical specimens and general information revealed that stage_pT (*P* = 0.015), stage_pN (*P* = 0.029), post-ER status (*P* = 0.010), post-Ki67 index (*P* < 0.001) were influencing factors for DFS, as determined through univariate and multivariate COX regression analysis (Table 3).

**Table 3:** Univariate and multivariate analyses of 5-year recurrence and patients’ post-NACT characteristics.

Characteristics	Univariate analysis HR (95% CI)	*P* value	Multivariate analysis HR (95% CI)	*P* value
Age, years (≥ 45 vs. < 45)	0.776 (0.467–1.291)	0.489		
Menopausal status (Postmenopausal vs. Premenopausal)	0.881 (0.683–1.136)	0.329		
pT stage		**0.019**		**0.015**
pT1	1 (reference)		1 (reference)	
pT2	1.002 (0.588–1.709)		1.109 (0.641–1.917)	
pT3/pT4	2.753 (1.299–5.836)		3.043 (1.402–6.603)	
pN stage		**0.025**		**0.029**
pN0	1 (reference)		1 (reference)	
pN1	1.601 (0.829–3.090)		1.723 (0.889–3.341)	
pN2/pN3	2.555 (1.287–5.072)		2.568 (1.280–5.150)	
Histological grade (III vs. I/II)	1.580 (0.871–2.866)	0.138		
post-ER status		**0.011**		**0.020**
Negative	1 (reference)		1 (reference)	
(1, 10]	1.162 (0.352–3.841)		0.916 (0.254–3.303)	
(10, 40]	0.204 (0.055–0.757)		0.198 (0.050–0.778)	
(40, 70]	0.367 (0.136–0.989)		0.358 (0.126–1.019)	
> 70	0.331 (0.134–0.815)		0.277 (0.106–0.727)	
post-PgR status		0.176		
Negative	1 (reference)			
(0, 50]	0.774 (0.442–1.355)			
> 50	0.538 (0.280–1.035)			
post-Ki67(%)		**0.003**		**< 0.001**
≤ 20	1 (reference)		1 (reference)	
(20, 50]	1.344 (0.757–2.388)		1.385 (0.775–2.474)	
> 50	3.053 (1.618–5.761)		3.825 (1.971–7.420)	
NACT regimens (EC-T vs. TEC)	0.758 (0.374–1.535)	0.441		

*DFS* disease-free survival, *HR* hazard ratio, *CI* confidence interval, *NACT* neoadjuvant chemotherapy, *post-ER* estrogen receptor after NACT, *post-PgR* progesterone receptor after NACT, *post-Ki67* Ki67 index after NACT.

Bold values indicates statistical significance (*P* < 0.05).

## Discussion

At present, neoadjuvant chemotherapy is extensively applied to reduce the disease stage of advanced breast cancer and enable the possibility of breast-conserving treatment. Our study presents retrospective real-world clinicopathological data and prognostic outcomes for patients with ER-positive, HER2-low breast cancer treated with NACT. As reported in previous studies, the proportion of patients with HER2-low breast cancer was greater in the ER-positive group than in the ER-negative group^10,16^. Additionally, our previous study showed that patients with ER-positive, HER2-low breast cancer exhibit poor sensitivity to NACT. In this cohort, the rates of achieving pCR between HER2-low and HER2-negative patients were significantly different (HER2-low: 11.9%, HER2-negative: 21.0%, *P* < 0.001)^12^. These findings are in line with those in other reports, for instance, Baez-Navarro et al.^17^ reported that patients with HR-positive, HER2-low breast cancer had the worst pCR rate (3.9%). Poor responsiveness to chemotherapy and the relatively high proportion of overall cases result in numerous cases of ER-positive, HER2-low patients with residual disease.

Although the prognosis of HER2-enriched breast cancer has been greatly improved through the application of anti-HER2 targeted therapy, it is still the subtype of ER-positive breast cancer with the worst prognosis^18,19^. Interestingly, extremely similar DFS rates were observed in the HER2-negative and HER2-low cohorts, which aligns with the findings in previous studies^9,10,20,21^. Subsequently, we examined the prognosis of HER2-low patients based on NACT efficacy. Consistent with other findings, patients with ER-positive, HER2-low breast cancer who did not achieve pCR after NACT had a significantly greater recurrence risk than those who achieved pCR (*P* = 0.020)^22,23^. To further explore the independent factors affecting recurrence in ER-positive, HER2-low patients with residual disease, we conducted a detailed analysis.

Pathological evaluation of primary disease and metastatic lymph nodes is highly important for predicting prognosis. Similarly, the presence of residual tumors reflects the sensitivity of tumors to chemotherapy. In our study cohort, although the pCR rate was only 11.9%, approximately half of the other patients had residual tumors with a maximum diameter of less than 2 cm, which sufficiently showed the importance of NACT in breast conservation strategies. After conducting a comprehensive analysis, we found that DFS was related to post-NACT tumor size (*P* = 0.015) but not to pre-NACT tumor size (*P* = 0.837). This suggests that the sensitivity of the tumor to chemotherapy, as indicated by the reduction in tumor size, has a relatively greater impact on prognosis. Notably, lymph node status is closely related to the risk of recurrence. Our prognostic analyses of both pre- and post-NACT data revealed that lymph node status was significantly correlated with recurrence. Likewise, several studies have demonstrated that post-NACT lymph node status has a substantial impact on prognosis^24–26^. For instance, Lee et al. reported that the prognosis in patients without lymph metastasis after NACT was not significantly different regardless of the lymph node status before NACT (*P* = 0.152)^24^. Overall, diseases with more advanced stages after NACT challenged the ability of the operator and were more inclined to recurrence.

In addition, the results of pathological analysis after NACT treatment revealed a noticeable reduction in malignancy, including decreases in the Ki67 index and tumor grade. The Ki67 index serves as an indicator of malignant proliferation, which is closely related to local recurrence and distant metastasis of breast cancer^27^. In our retrospective analysis of ER-positive patients with tissue available for Ki67 analysis, we found that patients with higher pre-NACT Ki67 levels were more likely to achieve pCR than those with lower pre-NACT Ki67 levels^14^. Furthermore, several studies have reported that a decreased Ki67 index after NACT is associated with favorable clinical outcomes^28–31^. Consistent with this, we found that both pre- and post-NACT Ki67 expression levels were associated with DFS, particularly highlighting the increased risk of relapse for patients with residual tumors with higher proliferation activity (*P* < 0.001). However, some studies have indicated that the post-NACT Ki67 level of residual tumors has independent prognostic significance, whereas the pre-NACT Ki67 level does not have the same significance^22,31–33^. In addition, there was no significant correlation between histological grade and DFS in our study, which may indicate that cancer cells scattered throughout the human body are the primary cause of recurrence^34,35^. Then, we observed variations in the expression levels of ER and PR after NACT. Heterogeneity is likely the reason for these differences, leading to distinct biological characteristics within the tumor^36^. Previous studies reported that NACT leads to a transformation between HR positivity and HR negativity, and 26 of the ER-positive patients experienced negative conversion after NACT in our study. Consistent with most findings, a higher expression level of ER was associated with a better prognosis^37,38^. Moreover, multivariate analysis suggested that even in patients in whom the ER expression level is positive after NACT, if it remains below 10%, the outcomes are similar to those of ER-negative patients.

Despite the extensive efforts made to enhance the rigor and dependability of this research, there are still several limitations. For instance, the number of included patients was restricted due to missing patient historical data or loss to follow-up, and our sample was insufficient for establishing a clinical prediction model and validating it. Furthermore, the characteristics of retrospective studies hinder the improvement of many clinically and pathologically significant features, such as the P53 mutation status. Our collection of the pre- and post-NACT prognostic factors cannot guarantee subjective consistency between the two sets. Additionally, this study involved only Chinese individuals, limiting the generalizability of the results to populations with diverse racial backgrounds. The single-center and retrospective design of the study may have resulted in both selection and information biases. Moreover, with the development of chemotherapy drugs, NACT may have greater efficacy than before, so our research can provide limited guidance for NACT in patients with low HER-2 expression in current clinical practice. It is worth mentioning that the pCR rates for both groups were higher than in our previous study conducted between 2012 and 2016. Consequently, with the development of chemotherapy medications, our investigation may provide limited guidance for the use of NACT in patients with ER-positive, HER2-low breast cancer in present-day clinical practice^12^.

## Conclusion

Previously, we found that ER-positive, HER2-low patients have low sensitivity to NACT; thus, we further analyzed the outcomes in these patients. We collected comprehensive patient data as much as possible to identify the factors affecting recurrence. We found that advanced disease stage, a greater Ki67 index and lower ER expression were associated with a superior five-year recurrence rate in ER-positive, HER2-low patients with residual disease after NACT. Patients with a high risk of recurrence will be candidates for new ADC drugs in the future.

## Data Availability

The datasets generated and/or analysed during the current study are available in the figshare repository, [10.6084/m9.figshare.22294111].
